# Integrated Amplification Microarrays for Infectious Disease Diagnostics

**DOI:** 10.3390/microarrays1030107

**Published:** 2012-11-09

**Authors:** Darrell P. Chandler, Lexi Bryant, Sara B. Griesemer, Rui Gu, Christopher Knickerbocker, Alexander Kukhtin, Jennifer Parker, Cynthia Zimmerman, Kirsten St. George, Christopher G. Cooney

**Affiliations:** 1 Akonni Biosystems, Inc., 400 Sagner Avenue, Suite 300, Frederick, MD 21701, USA; Email: lbryant@akonni.com (L.B.); cknickerbocker@akonni.com (C.K.); akukhtin@akonni.com (A.K.); jparker@akonni.com (J.P.); czimmerman@akonni.com (C.Z.); cooney@akonni.com (C.G.C); 2 Laboratory of Viral Diseases, Wadsworth Center, New York State Dept of Health, 120 New Scotland Avenue, Albany, NY 12208, USA; Email: sbg03@health.state.ny.us (S.B.G); rxg11@health.state.ny.us (R.G.); kxs16@health.state.ny.us (K.S.G.)

**Keywords:** microfluidics, diagnostics, gel element arrays, asymmetric PCR, RT-PCR, reverse transcriptase, multiplex, integrated microarrays

## Abstract

This overview describes microarray-based tests that combine solution-phase amplification chemistry and microarray hybridization within a single microfluidic chamber. The integrated biochemical approach improves microarray workflow for diagnostic applications by reducing the number of steps and minimizing the potential for sample or amplicon cross-contamination. Examples described herein illustrate a basic, integrated approach for DNA and RNA genomes, and a simple consumable architecture for incorporating wash steps while retaining an entirely closed system. It is anticipated that integrated microarray biochemistry will provide an opportunity to significantly reduce the complexity and cost of microarray consumables, equipment, and workflow, which in turn will enable a broader spectrum of users to exploit the intrinsic multiplexing power of microarrays for infectious disease diagnostics.

## 1. Introduction

While every infectious disease presents specific diagnostic challenges, there are several themes that emerge from clinical needs that eventually impact the design and development of diagnostic technologies themselves. These include but are not limited to: the number and types of microorganisms that may result in a particular assemblage of symptoms; an abundance of phylogenetically or phenotypically related microorganisms on or within the human host, and in the local (non-human) environment; rapid evolution and the acquisition of new traits, either through mutation or horizontal gene transfer; an ability to move or survive between and within the human host, non-human vectors, and/or environmental reservoirs; the capacity for colonization or dormancy without causing disease; and an aptitude to be transmitted or cause disease at low levels of infection. Even if an infection is not life-threatening, there are some universal principles and clinical user needs associated with sensitivity, specificity, total analysis time, and ease of use that have technical implications for a diagnostic platform.

Diagnostic tests based on metabolic activity (e.g., growth or secondary metabolites) or molecular building blocks (e.g., lipids, proteins, nucleic acids) are developed for a very specific clinical context. Of these, nucleic acid tests based on real-time polymerase chain reaction (PCR) technology address many of the sensitivity and specificity challenges for infectious disease diagnostics, leading to widespread adoption of nucleic acid diagnostics in surveillance, epidemiology, and clinical practice over the last decade (e.g., [1,2,3,4,5,6,7,8,9,10,11]). The advent of integrated PCR systems [12,13,14,15] that address user needs for rapid analysis times and ease-of-use are likewise expected to expand and accelerate the adoption of molecular diagnostics in clinical practice, including point-of-care and point-of-use settings [16,17,18,19]. Integrated real-time PCR systems, however, typically comes at the cost of mid- to high-level multiplexing, or the ability to detect multiple microorganisms, nucleotide polymorphisms or drug resistance mutations from a single sample.

Microfluidic PCR systems tend to address the multi-analyte detection problem by splitting a purified nucleic acid sample into spatially isolated analysis channels or reaction wells, where each analysis chamber contains target-specific primers and detection probes (e.g., Idaho Technologies FilmArray). Some of these systems can now support thousands of discrete real-time PCR tests in a single run (BioTrove OpenArray, and related life sciences tools). A split assay is applicable provided that the purified, target nucleic acids can be subdivided but still amplified and detected by the end-point detector (*i.e*., not split into extinction). The manual or automated manipulations intrinsic to parallel amplification systems, however, are still subject to a basic requirement for ≥10^2^ copies per reaction well or vessel to avoid molecular sampling error [20,21,22] and its potential to cause false negative results, unless one employs a nested PCR strategy (as in the FilmArray). Splitting a sample (or target DNA) to extinction is actually desirable in limiting dilution PCR [23], which has now been translated into digital PCR [24], microfluidic chips, and various commercial products (e.g., Fluidigm Digital Array, BioRad ddPCR). While digital PCR is currently used for primarily quantifying DNA and analyzing copy-number variations, multiplexed digital detection is on the horizon [25], as are isothermal digital PCR systems [26]. In contrast to these PCR technologies, microarrays interrogate hundreds to millions of genetic signatures across multiple genes in a homogenous assay, and have therefore emerged as a potentially useful diagnostic platform when sample or total nucleic acid concentration is limiting [2,27,28,29,30,31]).

A typical microarray workflow may involve numerous steps, including nucleic acid purification, nucleic acid amplification (gene-specific, or whole genome amplification), a second round of amplified target purification, target fragmentation, target labeling, possibly a third round of target purification, dilution with hybridization buffer, target hybridization, microarray washing, imaging, and data analysis (see e.g., [32,33,34,35,36,37,38]). In the absence of a fully automated and enclosed system and because of a continued reliance on amplification chemistry, there is significant potential for cross-contamination between samples, or contamination of the workspace with amplified nucleic acids. These workflow concerns were a primary impediment to the clinical adoption of PCR, a situation that was only surmounted with the advent of real-time PCR. In addition, the hybridization and detection steps may be further complicated depending upon whether or not the test uses allele-specific extension, ligation, or signal amplification chemistries. Some microarray methods have been fully automated, either as prototype instruments or commercial products ([39,40,41,42,43,44,45]; see also products from Autogenomics, Nanosphere; ClonDiag, and Luminex). However, microarray sensitivity and specificity are maximized with extended (>16 h) hybridizations that drive the hybridization reactions to thermodynamic equilibrium, which is generally inconsistent with the requirements for an infectious disease diagnostic test. Re-circulating flow and sample agitation are some of the methods used to improve hybridization kinetics and reduce total hybridization time, which either adds to the equipment infrastructure needed to perform a microarray test, or adds to the complexity (and cost) of an integrated microarray system. Thus, microarrays have not yet found widespread use beyond the research community due to labor, time intensive protocols, instruments and analysis software that typically require very advanced training to operate or interpret. 

Several commercial microarray manufacturers have simplified microarray work flow by engineering fluidic hybridization, washing, and imaging stations, and providing semi-automated software and data analysis. Sample preparation and amplification chemistries are also now being incorporated into the workflow either through robotic [43,46] or microfluidic transfer steps [15,41,44,47,48,49,50]. In spite of these advances, engineering and manufacturing challenges scale with the complexity of the integrated, microfluidic microarray systems, while robotic systems must contend with an open-amplicon workflow that plagued the early adoption of PCR technologies in clinical practice. An alternative approach to simplifying microarray workflow, especially for lower-resource settings, is to re-evaluate the biochemistry and processing steps that precede and interface with the microarray itself. By simplifying the biochemistry and analytical steps in the process, there is a corollary opportunity to simplify the complexity and cost of the microarray-based consumables and instrumentation, while meeting user needs for total analysis time, sensitivity, and specificity. This overview summarizes recent methods for combining target amplification, labeling, and microarray hybridization into a single, closed-amplicon reaction chamber, and provides examples of an integrated biochemical approach for amplification array-based analysis of DNA and RNA genomes. We also illustrate a simple consumable architecture for incorporating post-amplification wash steps with an amplification microarray while retaining an entirely closed system, which exemplifies one approach to a consumable architecture for producing low-cost, easy-to-use, closed-amplicon microarrays for point-of-use applications.

## 2. Solid Phase Amplification

Early attempts to combine target amplification and array detection involved amplifying target nucleic acids on a solid support, where the amplification primers were cross-linked to a surface [51,52,53,54,55,56,57,58]. In most cases, these experiments showed that solid-phase PCR is less efficient than conventional solution-phase reactions with relatively poor limits of detection (approximately 10^5^–10^6^ genomes per reaction). These limits of detection have significant negative implications for infectious disease diagnostics. Supplementing the reaction mixture with unbound, gene-specific primers and allowing the PCR to simultaneously proceed in the liquid and solid phases was one approach to increase product yield and analytical sensitivity [59,60,61,62,63,64]. These schemes represent an analog of nested PCR, where the first stage of the reaction amplifies the nucleic acid of interest in solution using free-floating primers, and the second stage results in attachment of amplified fragments to immobilized PCR-primers with subsequent chain extension. As with any nested PCR procedure where the products of the first amplification phase are not purified before the second amplification phase, however, primer artifacts and primer interference tended to restrict multiplexing capacity and amplification efficiency.

More recent variants of solid-phase or microarray-based PCR are described in several publications. For example, Tillib *et al.* [62] attempted to overcome primer interference by creating a microarray of monoplex PCR chambers separated from each other by mineral oil. Pemov *et al.* describe a gel element array where multiplex PCR occurs on and within gel element arrays and is enhanced by pseudo-monoplex PCR in solution [65]. Li *et al.* [66], designed a microarray of hydrophilic microwells patterned on a hydrophobic chip, where primer pairs tagged with a universal sequence were physically separated in the individual hydrophilic microwells. This construct enabled many unique PCR reactions to be proceeded simultaneously during the first step of the procedure, similar to the approach described by Tillib [62]. However, Li *et al.* isolated the first-stage amplification products from the PCR array for subsequent analysis by gel electrophoresis or conventional DNA microarray. Sun *et al.* describes an approach to influenza RNA amplification and detection, where RNA is reverse transcribed in solution over the biochip, PCR is initiated in solution with free-floating reverse primers, and the resulting cDNA is then extended from nested, immobilized primers on the array [67]. A related method used gene-specific, immobilized reverse primers to directly interrogate the mRNA transcriptome of mouse muscle fibroblasts [68]. Isothermal, helicase-dependent, solid-phase amplification has also been demonstrated, which provides an opportunity to simplify the attendant instrumentation by eliminating the need for a thermal cycler [69]. Unfortunately, solid-phase amplification is limited by the kinetics of (low-copy) target hybridization to the array surface during the initial rounds of the amplification reaction.

## 3. Integrated Biochemistry for Single-Step, Closed-Amplicon Microarrays

There are reports of highly multiplexed, solution-phase amplification techniques that precede microarray detection, including isothermal amplification reactions, whole genome amplification systems, and highly multiplexed, gene-specific reactions (e.g., [70,71]). The underlying principles rely on driving the amplification reaction to the plateau phase and using microarray hybridization to detect specific amplicons from amplification artifacts that may arise as a consequence of the high multiplexing.

**Figure 1:**
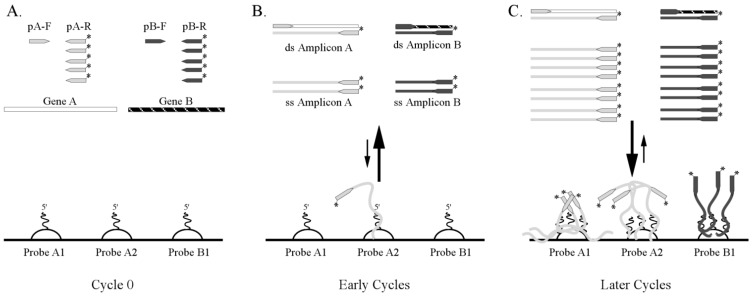
Integrating amplification and hybridization chemistry within an amplification microarray and single microfluidic chamber. (**A**) Gene-specific reverse amplification primers (pA-R and pB-R) are labeled with a fluorophore and provided in excess relative to the forward primers (pA-F and pB-F). The microarray may contain one or more probes for each target gene (GeneA and Gene B) and resulting amplicon. Two probes are shown for Gene A and one probe is shown for Gene B. (**B**) The initial rounds of the amplification create both double-stranded and single-stranded amplicon, but hybridization to the microarray is kinetically limited because single stranded amplicon has not yet accumulated. (**C**) Towards the final rounds of amplification, single stranded amplicons abound, so hybridization to the microarray is kinetically favorable. Hybridization times can be extended beyond the amplification reaction to achieve thermodynamic equilibrium, if desired.

In one incarnation of this approach, microarrays are printed within a gel-lined cap of a microcentrifuge tube. Nucleic acid amplification occurs within the bottom of the tube, and after amplification the tube is inverted, a hybridization buffer is released from an internal chamber, and microarray hybridization occurs within the PCR vessel [72]. The array tube format is intrinsically user friendly, but the limited surface area of a microcentrifuge cap constrains the microarray-based multiplexing capacity of the test and the arrays cannot be washed without removing the cap. A fully integrated, single cartridge for PCR amplification, array hybridization, signal enhancement, washing, and imaging has recently been described for *in situ* synthesized arrays [41], but the system uses off-board reagents and a fluidic processing station to complete all processing steps. The general concept of integrating quantitative, real-time PCR with microarray readouts has likewise been reported. Khodakov *et al.* used a first-stage, multiplex PCR in solution, and then performed a real-time, quantitative PCR amplification on gel element arrays [73]. SYBR Green I dye intercalation was used to detect target nucleic acids that were extended from gel-immobilized primers during the final 3 sec of the elongation step. Pierik *et al.* [74] describe a similar system and approach, except that a Cy5 fluorescent tag is incorporated into one of the solution-phase amplification primers, and real-time quantitation is based on target hybridization to the array rather than chain extension from the array. In this case, only a single-plex reaction was demonstrated. In both cases, customized instruments that integrate a thermal cycler with optical detection around a planar substrate are required, the amplification efficiency from the array is rather low, and relatively large number of cycles are required to achieve limits of detection that are useful for infectious disease diagnostics.

From the foregoing literature review, it is clear that the development of closed-amplicon, microarray-based diagnostics still requires advances in integrated biochemistry, microfluidics, hardware, and software to meet user needs and requirements of infectious disease diagnostics. Towards that end, we are exploiting advances in solution-phase multiplexed amplification chemistry, the solution-phase properties and high probe immobilization capacity of gel element arrays (e.g., [75]), and microfluidic consumables [76] to significantly simplify microarray workflow for infectious disease diagnostics. The principles for simplifying the biochemical steps are to utilize multiplex, asymmetric PCR or reverse-transcriptase PCR in solution with Cy3-labeled “reverse” primers, perform thermal cycling in the presence of the gel element array, and allow the predominantly single-stranded, labeled amplicons to hybridize to the array during (or after) on-chip thermal cycling (Figure 1). Microarrays are washed (after thermal cycling) in bulk solution or with a bolus of self-imbibing wash solution pipetted into a flow cell that contains an integrated waste chamber. Three examples of the integrated biochemistry approach and one method for integrating wash steps within an amplification microarray consumable are illustrated here. Specific technical details for each of the tests can be provided upon request.

### 3.1. One-Step RT-PCR Amplification Microarray

Influenza viruses are highly contagious, segmented, negative-sense RNA viruses that cause approximately 114,000 hospitalizations and 20,000 deaths in the U.S. annually [77]. Seventeen hemagglutinin (HA) subtypes and 10 neuraminidase (NA) subtypes are recognized, although until recently only viruses of the H1N1, H2N2, and H3N2 subtypes have been associated with widespread epidemics in humans [78,79]. Both HA and NA glycoproteins undergo antigenic drift as a result of sequence changes driven by multiple evolutionary pressures. Antigenic shift occurs when viral RNA segments re-assort during co-infection of cells by different influenza A subtypes. Rapid influenza tests are generally unable to identify influenza strains that have undergone shift or drift, or determine the match between circulating influenza viruses and those viruses contained in vaccines. Surveillance data from more sophisticated tests are therefore needed to monitor for the emergence of antiviral resistance or new influenza A subtypes that might pose a pandemic threat. The diagnostic challenge, then, is for a test to detect many different influenza signatures in trace amounts, from relatively complex and diverse sample matrices and biological backgrounds.

PCR primers and microarray probes were designed from real-time RT-PCR assays developed and used by the Laboratory of Viral Diseases at the Wadsworth Center, New York State Department of Health. Gel element microarrays were manufactured essentially as described in [80] and targeted various portions of the M, NS, HA, NP and NA genes. Control probes include Cy3 beacons for positional reference, and a human GAPDH internal positive control for sample collection, extraction, reverse transcription, amplification, and detection. The microarray was surrounded by a single 50 uL gasket, and the RT-PCR amplification microarray master mix was applied to the RT-PCR amplification microarray, followed by RNA template. Arrays were sealed with a plastic cover slip and the microarray substrates mounted on a flat block thermal cycler, processed for 40 thermal cycles, and then hybridized at room temperature for up to 2 h. There was no post-PCR target labeling, fragmentation, purification, quantitation, or transfer into hybridization buffer, as typically required for conventional microarray procedures. After hybridization, the cover slips were removed, microarrays transferred to a histology slide holder, and then gently washed in buffer. Slides were then dipped in de-ionized water, air dried, and imaged on an Akonni portable, prototype imager with a 0.5 s exposure time. Local background was subtracted from the signal of each gel element and integrated, background-corrected signal intensity data exported into Excel spreadsheets for data analysis. Signal was calculated as the median background-corrected integral intensity for each probe, where each probe was printed in quadruplicate per array. Noise was calculated as 3 times the average standard deviation of all local backgrounds. Signal-to-noise ratios (SNR) >3 were considered detectable over all sources of noise. The total analysis time for the prototype RT-PCR amplification microarray test was approximately 6 h using a 2 h post-PCR hybridization time.

**Figure 2:**
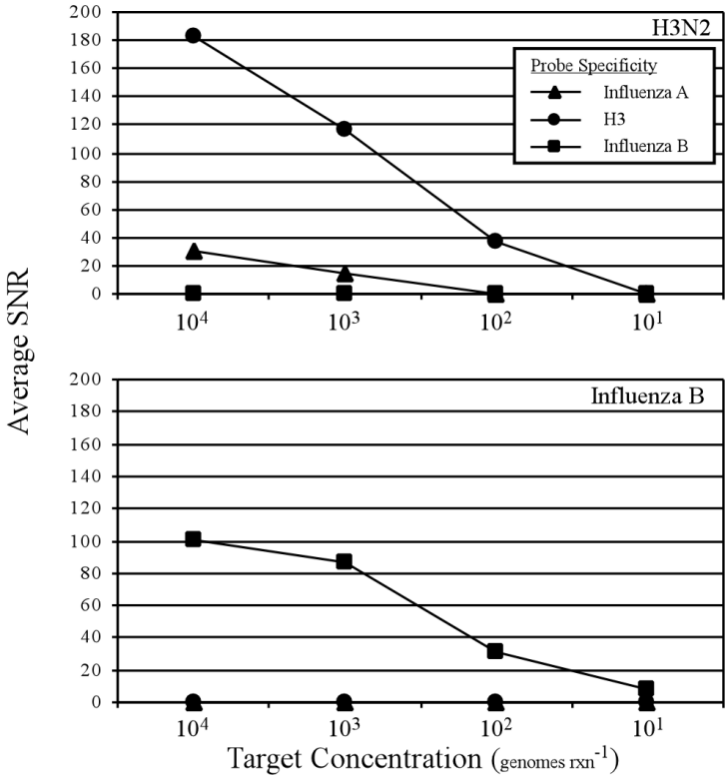
Sensitivity and specificity of a prototype influenza RT-amplification microarray. Results are the average from two technical replicates, where positive detection is defined as a signal to noise threshold ≥3.

Estimated limits of detection and amplification microarray specificity for influenza A/H3N2 and influenza B and their respective, perfectly-matched target probes are shown in Figure 2. The influenza A/H3 probe was detectable at an input of 10^2^ genomes per reaction while the influenza A probe was detectable at 10^3^ genomes per reaction. In contrast, influenza B was readily detected at 10 genomes·rxn^−1^. These results are consistent with those obtained with a conventional microarray amplification and hybridization approach and identical microarray probes (not shown), and demonstrate the potential for sensitive influenza detection within a single-step, closed amplicon reverse transcriptase amplification microarray. The bulk washing procedure described here was used primarily to increase throughput during the development and optimization of the multiplex RT-PCR and integrated hybridization chemistry, and such a manipulation may or may not be an acceptable practice in some clinical work environments or low-resource settings. One possible solution to this challenge is a valve-less, integrated flow cell that incorporates an inlet port and waste chamber, as described in detail in [76] and illustrated below.

### 3.2. Two-Step RT-PCR Amplification Microarray for DNA and RNA Genomes

Encephalitis and meningitis are potentially fatal diseases defined by acute inflammation of the brain or protective membranes covering the brain and spinal cord of the central nervous system. These diseases can be caused by viruses, bacteria, fungi and parasites, and disproportionately affect children, the elderly, or the immunocompromised. Viruses are the most common cause of encephalitis, with the majority of aseptic encephalitis cases caused by enteroviruses, human herpesviruses, and arboviruses. Unfortunately, clinical presentation and initial laboratory findings in most cases of meningitis and encephalitis are overly nonspecific to permit an etiologic diagnosis. Because complications arising from CNS infection and appropriate treatment strategies can often depend on the organism involved, it is important to differentiate between those cases for which a specific treatment has been shown to be effective, and those for which only supportive treatment is indicated.

PCR primers and microarray probes for herpes simplex viruses 1 and 2 (HSV-1, HSV-2), varicella zoster virus (VZV), cytomegalovirus (CMV), human herpesvirus 6 (HHV-6), enterovirus (EVU) and West Nile virus (WNV) were designed from real-time PCR assays originally developed and used by the Laboratory of Viral Diseases at the Wadsworth Center, New York State Department of Health. Control probes included Cy3 beacons for positional reference, and the green fluorescent protein (GFP) gene as an internal positive control for amplification and detection. In this case, the multiplex, asymmetric master mix consists of 8 primer pairs. Viral RNA, DNA and cDNA templates were quantified by clinically validated real-time PCR assays before use. Reaction mixtures were assembled and transferred to the gel element array chambers as described above. Amplification microarrays were processed for 45 cycles, and then hybridized at for 1–3 h, with no user intervention after applying the amplification master mix to the microarray. After hybridization, microarrays were washed, dried, and imaged as described above. The total analysis time for a test that included a 1 h, post-PCR hybridization was approximately 4 h.

**Table 1:** Average signal to noise ratios (n = 2) for a multiplex, closed-amplicon amplification microarray. Bold type indicates specific amplification microarray signals.

Target (gene copies per reaction)								
Probe Specificity	HSV1 (5,000)	HSV2 (500)	VZV (500)	GFP (5,500)	EVU (500)	CMV (500)	HHV6 (500)	WNV (500)
HSV1	**34.56**	0.72	0.67	0.44	0.68	0.55	0.54	0.17
HSV2	1.70	**66.94**	0.14	0.21	−0.08	0.16	0.64	−0.01
VZV	0.86	0.64	**24.82**	0.76	0.44	−0.21	0.90	0.05
GFP	0.76	1.10	0.43	**244.26**	0.52	0.53	0.04	0.21
EVU	1.03	0.57	0.63	0.27	**198.73**	−0.03	1.21	0.14
CMV	0.81	0.92	0.57	0.62	4.31	**346.28**	0.36	0.20
HHV6	1.65	0.67	1.16	0.22	0.72	0.70	**132.13**	−0.31
WNV	0.74	0.64	0.75	0.34	0.65	0.52	1.72	**472.55**

Results for 500 to 5,000 gene copies of each nucleic acid target per reaction are shown in Table 1. For these experiments, EVU and WNV RNA were first reverse-transcribed with random primers for 60 min before adding cDNA to the amplification microarray. Amplification and detection specificity was 100%. No template controls were all negative (not shown). When the same two-step asymmetric, multiplex amplification microarray was tested against >10^5^ gene copies of non-target microorganisms, there was no detectable signal and no visible amplification product as determined by agarose gel electrophoresis (not shown). While a test-tube reverse transcriptase step (Table 1, RNA viruses) does not evoke the same level of cross-contamination concerns as transferring amplified material from one reaction tube to another, the user work flow is certainly simplified with a single-step RT-PCR amplification microarray procedure. Table 2 shows results for single-plex, on-chip, asymmetric reverse transcriptase amplification microarray detection of EVU and WNV RNA to at least 100 copies per reaction, an analytical sensitivity comparable with conventional, test tube, one-step RT-PCR.

**Table 2:** One-step, asymmetric RT-amplification microarray detection of West Nile virus and enterovirus RNA in single-plex reactions. Data are the average signal to noise ratios from n = 3 technical replicates. Bold type indicates specific amplification microarray signals. No template controls were all negative (not shown).

Target (gene copies per reaction)						
Probe Specificity	Enterovirus RNA			West Nile virus RNA		
	10^4^	10^3^	10^2^	10^4^	10^3^	10^2^
HSV1	0.21	0.05	0.09	0.18	0.39	0.12
HSV2	−0.25	0.08	−0.08	−0.46	−0.20	−0.30
VZV	0.76	0.38	0.39	0.20	0.07	−0.01
GFP	−0.25	0.25	0.01	0.13	−0.27	0.07
EVU	**405.00**	**198.73**	**29.57**	−0.16	0.08	−0.44
CMV	3.49	1.38	0.74	0.13	0.15	0.11
HHV6	0.03	0.57	0.11	0.27	0.22	−0.35
WNV	−0.26	0.03	0.01	**1,093.61**	**824.32**	**1,263.02**

### 3.3. Integrated Waste Chamber and Entirely Closed-Amplicon Consumable

Conventional drug susceptibility testing of *M. tuberculosis* isolates may take weeks or months, and while molecular methods are more rapid, there are numerous genes and single nucleotide substitutions within those genes that confer antibiotic resistance (see review of multi-drug resistant TB mutations in [81]). In 2006, approximately 5% of all new TB cases were MDR, an increase of 12% since 2004 and a 56% increase since 2000. From 2008 until 2010, the number of global MDR-TB cases grew an astounding 48% from 440,000 to 650,000 [82]. Molecular tests are therefore needed that can span the totality of known drug resistant mutations for both epidemiological investigations and clinical diagnostics.

Low-resource settings, where tuberculosis is a significant public health problem, present some technological, manufacturing, and operational challenges for multiplexed tests. Cold-chain storage, intermittent power, complex workflows, challenging sample matrices (*i.e*., sputum), and biosafety concerns are several of the operational realities that exist in these locations. Even a relatively simple post-amplification wash as described above is operationally problematic. While integrated systems overcome a number of these challenges, many of the integrated, microfluidic, array-based detection systems that are expensive to manufacture and operate, and often require significant financial subsidies to reach affordable levels for governments and users where TB is endemic. For these and related reasons, then, there is now significant interest in developing “resource-appropriate” molecular platforms for TB and other infectious diseases.

**Figure 3:**
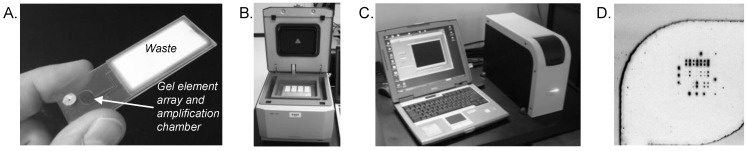
(**A**) Closed-amplicon amplification microarray with integrated waste chamber. (**B**) Amplification microarray flow cells in a Quanta flat block thermocycler. (**C**) Akonni portable microarray analyzer used to image amplification microarrays. (**D**) Image of a prototype MDR-TB array following a 50-cycle closed-amplicon asymmetric PCR protocol.

We have developed a valve-less, integrated amplification microarray consumable that addresses some of these work-flow and operational concerns [76]. The simplifying fluidic principle uses some of the concepts of lateral flow devices for post-PCR washing and drying the array chamber. Samples are introduced into the integrated flow cell with a pipette, thermal cycled, and then washed by injecting wash solution through the amplification/array chamber. All solutions (unbound amplicons, wash solutions) are automatically imbibed into the waste chamber, mimicking lateral flow fluidics. The feasibility for such a valve-less amplification microarray and consumable is shown here for a prototype MDR-TB microarray. Primers and probes were included for *rpoB* (five mutations), *katG* (one mutation), *inhA* (four mutations), IS6110 (*M. tuberculosis* complex), and IS1245 (*M. avium *complex). A matched pair of microarray probes (wildtype and single-nucleotide mutant) was designed for each mutation of interest. Nucleic acids from NALC-NaOH treated, heat-killed *M. tuberculosis* were isolated and assembled into a 5-plex, asymmetric amplification microarray reaction mixture before introducing the solution through the sample inlet port (Figure 3(A)). The port was then sealed with a foil disk, the array processed on a flat block thermocycler (Figure 3(B)) for 50 cycles, and then hybridized for 3 h. After thermal cycling and hybridization, the array chamber was washed by piercing the inlet port with a pipettor and sequentially dispensing buffer, Milli-Q water, and acetone through the chamber. Microarrays were imaged on Akonni’s portable analyzer (Figure 3(C,D)), and analyzed as described above. None of these experiments required disassembly of the flow cell or reaction chamber (as for the influenza and encephalitis examples described above), thereby maintaining a truly closed-amplicon workflow that is appropriate for lower-resource settings and most CLIA-certified molecular diagnostic laboratories.

**Table 3:** Amplification microarray flow cell genotyping of MDR and wild-type M. tuberculosis samples (250 pg and 15 pg). Wild-type to mutant probe ratios <1 (bold, shaded) indicate a mutation at the designated position within the gene(s) of interest.

DST Phenotype and DNA amounts		MDR 250 pg	MDR 15 pg	WT 250 pg	WT 15 pg
**Gene**	**Mutation**	**Wildtype to Mutant Ratios**			
***rpoB***	**D516V**	5.8	9.1	5.4	8.4
	**H526D**	3.5	7.9	4.4	8.5
	**H526Y1**	3.9	5.1	2.7	3.4
	**S531L**	**0.1**	**0.1**	2.2	3.1
	**L533P**	9.1	7.9	7.2	13.4
***katG***	**S315T**	**0.2**	**0.3**	12.0	11.5
***inhA***	**T8A**	9.1	4.9	8.2	4.5
	**T8C**	5.1	2.8	4.7	2.5
	**C15T**	7.0	7.7	7.4	7.8
	**G17T**	8.0	3.1	7.1	2.9

Genotyping results for two isolates (250 pg and 15 pg) are shown in Table 3 relative to the MDR-TB drug susceptibility phenotype, where a wild-type to mutant probe ratio >1 is indicative of a wild-type sequence at that gene position (hence, drug susceptibility), and ratios <1 are indicative of a drug-resistance mutation at that gene position. The isolates were correctly typed and the genotyping results were consistent with a more traditional procedure that involves test-tube multiplex PCR amplification followed by stand-alone, microarray hybridization (not shown). Similar on-chip PCR results were obtained with both 15 pg genomic DNA per reaction (~3.5 × 10^3^ genomes) and 250 pg (~5.8 × 10^4^ genomes). From this basic prototype, we can now significantly expand the microarray content to include all known *rpoB*, *katG* and *inhA* mutations that reside between the primer sequences, begin incorporating additional gene targets associated with antibiotic resistance to first- and second-line drugs, and optimize reaction conditions to genotype MDR-TB from as few as 10 genomes.

## 4. Discussion and Future Prospects

The example amplification microarrays described here illustrate that multiplex reverse transcriptase or PCR-to-microarray tests can be significantly simplified through integrated biochemistry and still achieve clinically relevant limits of detection. This represents a significant advance in clinical ease-of-use for microarray-based diagnostics. Translating the prototype tests into clinically useful research or diagnostic tools will require continued development of appropriate controls, prospective analysis of clinical samples, and a number of formal analytical studies to establish limits of detection and repeatability across arrays, users, and laboratories. Nevertheless, integrated biochemistry enables the development and manufacture of simple, low-cost consumables that can be integrated into current clinical environments and workflows without new infrastructure or expensive equipment. The extent of multiplexing for an amplification microarray appears to be dictated by the number of primer pairs in the master mix rather than the number of probes on the array, for the same reasons that tend to limit the number of genes that can be co-amplified by conventional solution-phase, multiplex PCR. Whether or not whole-genome amplification (WGA), multiple displacement amplification (MDA), or related approaches can be utilized within the context of an amplification microarray, the confines of a single chamber, and a single buffer condition is to be determined.

Whether a microarray is used independent of the amplification reaction, for solid-phase PCR, or as an amplification microarray, limits of detection are primarily governed by the interrelationship between starting template concentration, amplification efficiency, microarray hybridization kinetics, and the total number of amplification cycles. At low target concentrations that typify the early cycles of a solid-phase PCR or amplification microarray test, hybridization kinetics are slow. Even after amplicons begin to accumulate in solution over the biochip, microarray hybridization is most sensitive and specific at thermodynamic equilibrium (which is typically reached at >16 h, and with mixing/agitation). Because of thermodynamic and kinetic constraints of solid-phase hybridization, “rapid” microarray tests (inclusive of gel element arrays) therefore often come at the potential expense of detection sensitivity or specificity.

One approach to this kinetic constraint is to increase the total number of amplification cycles in order to “drive” microarray hybridization to completion, a method that is especially attractive for asymmetric (or log-linear) amplification reactions (see [83] for an 85-cycle example). In this case, the total analysis time is primarily based on the speed of thermal cycling and heat transfer rates to the on-chip solution. Flat block, peltier-driven, *in situ* thermal cyclers such as the one used here are much slower than the rapid cycling available in today’s most advanced tube-based thermal cyclers, and are unlikely to achieve 40–60 cycles within a 1–2 h time frame. Overcoming this limitation for PCR microarray-based consumables will therefore require new thermal cycler designs that conform to the unique shapes, sizes and heat-transfer properties of microfluidic, array-based consumables. In this regard, translating to thinner microarray substrates is expected to improve heat transfer rates and thermal cycling efficiency, so that the rate limiting step for amplification microarrays becomes enzyme processivity rather than thermal cycling ramp rates.

A potential biochemical approach to the thermal cycling challenge is isothermal amplification methods (e.g., [13,84,85,86,87,88,89]). Isothermal amplification systems are especially attractive for low-resource settings, but currently available enzymes have relatively slow processivity, the primer design strategy is complicated and not conducive to higher-levels of multiplexing, or the methods require multiple steps that would otherwise lead to a complex and expensive microfluidic consumable. Advances in basic enzymology or assay conditions are therefore needed to develop single-step isothermal methods that exploit the multiplexing power of microarrays. In the interim, we have demonstrated that single-step, integrated, closed-amplicon gel element microarrays that are as simple and uncomplicated as many real-time PCR tests can address microarray work flow challenges and clinically relevant infectious disease diagnostic problems.
